# Supramolecular Additive Screening to Engineer Microfibrous Rafts for Expansion of Pluripotent Stem Cells in Dynamic Suspension

**DOI:** 10.1002/adhm.202404186

**Published:** 2025-03-10

**Authors:** Johnick F. van Sprang, Jasper G. M. Aarts, Boris Arts, Joyce E. P. Brouns, Muhabbat I. Komil, Paul A. A. Bartels, Patricia Y. W. Dankers

**Affiliations:** ^1^ Institute for Complex Molecular Systems and Department of Biomedical Engineering Eindhoven University of Technology Eindhoven 5612AZ The Netherlands

**Keywords:** biomaterial, electrospinning, hiPSC, pluripotency, screening, supramolecular

## Abstract

Human induced pluripotent stem cells (hiPSCs) hold the potential to generate any human tissue for transplantation in regenerative therapies. These complex cell therapies require billions of cells, which is challenging to acquire in planar adherent cultures. Transitioning hiPSCs to 3D suspension culture on microcarrier materials, often bead‐shaped, improves the total surface area accessible to cells, thereby enabling culture scale‐up. However, bead‐shaped microcarriers do not have the optimal shape configuration, because it is the lowest surface‐to‐volume ratio of all geometrical shapes, and it also induces uncontrolled cell clumping. Application of synthetic, microfibrous rafts as a replacement for bead‐shaped microcarriers potentially solves these issues. Here, microfibrous rafts are engineered by first screening a supramolecular biomaterial library composed of bisurea (BU)‐peptide conjugate additives for its ability to induce hiPSC adhesion and maintenance of its pluripotent state, followed by electrospinning the screening‐hit into raft‐like structures. The resulting rafts contain cylinder‐like microfibers, which have a higher surface‐to‐volume ratio compared to conventional bead‐shaped microcarriers, and the flat configuration of the rafts prevents clumping.

## Graphical abstract

Segmented PCL‐bisurea (BU) polymers and BU‐peptide conjugate additives assemble into fibrous superstructures at the nanoscale through a mix‐and‐matching strategy. Different bioactivities are incorporated through variation of peptide sequences. The resulting materials are screened for their effect on stem cell adhesion and pluripotency. Screening hits are electrospun into microfibrous rafts and applied as scaffolds for dynamic suspension culture of pluripotent stem cells.

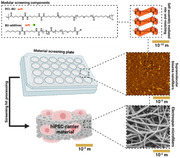

## 1 Introduction

Human‐induced pluripotent stem cells (hiPSCs) hold great translational promise due to their ability to theoretically generate any tissue type of the human body. These “synthetic” stem cells are acquired by genetically reprogramming adult somatic cells and afterward have the capacity to self‐renew indefinitely.^[^ ^1^, ^2^, ^3^ ^]^ The self‐renewal ability and rapid proliferation rate of hiPSCs allow for large‐scale expansion with the possibility of differentiation toward transplantable, in vitro‐generated tissue.^[^ ^4^, ^5^, ^6^ ^]^ It is estimated that >1 billion cells are required for a single regenerative therapy to treat muscular dystrophy, spinal cord injury, ischemic heart failure, or retinal damage.^[^ ^7^, ^8^, ^9^, ^10^ ^]^ Innovation is required in culture procedures and biomaterials for stem cell expansion to realize the clinical potential of hiPSCs. A combination of automated expansion systems, culturing of cells in a 3D geometric fashion, and synthetic biomaterials is expected to drastically improve the rate and ease of hiPSC expansion.^[^ ^11^, ^12^ ^]^

Originally, hiPSCs were grown on mouse‐embryonic feeder layers.^[^ ^1^ ^]^ In recent years, a switch has been made toward protein‐based coatings such as Matrigel.^[^ ^3^, ^13^, ^14^ ^]^ The current golden standard is widely considered to be recombinant protein‐based coatings such as vitronectin, and laminin.^[^ ^14^, ^15^, ^16^, ^17^ ^]^ However, natural‐derived materials suffer from poor processability and stability under non‐physiological conditions, limiting their application in production of microcarriers. This is due to the susceptibility of extracellular matrix (ECM)‐derived proteins to denaturation, which decreases their biological activity.^[^ ^18^ ^]^ Synthetic polymers are an alternative to these types of materials, circumventing the above‐mentioned limitations.

Numerous works have recently demonstrated that modification of synthetic polymers with short, ECM‐derived peptide sequences also allows for hiPSC adhesion and expansion.^[^ ^19^, ^20^, ^21^ ^]^ Synthetic materials have also been commercialized for this goal (e.g., Synthemax II, which is poly(acrylate) modified with RGD‐containing peptides).^[^ ^11^, ^22^ ^]^ These synthetic materials may also be used for coating of bead‐shaped microcarriers for hiPSC suspension culture. However, a bead (i.e., a sphere) has the lowest surface‐to‐volume ratio of all geometric shapes, indicating that the volumetric area is not completely exploited using such culture platforms. Instead, cylindrical‐like structures are more ideal geometric shapes with regard to accessible surface material relative to bulk volume. Another major disadvantage of using bead‐shaped microcarriers in hiPSC suspension culture is that it induces clumping behavior, which results in excessively large and uncontrolled hiPSC‐microcarrier aggregates.^[^ ^23^, ^24^, ^25^, ^26^, ^27^ ^]^ A cell carrier platform with a flat sheet mesh configuration, we henceforth term raft, is expected to avoid this clumping behavior while maintaining the advantage of a 3D suspension culture. By processing materials via electrospinning, it is possible to achieve raft‐like structures with fibrous microstructure.

**Figure 1:**
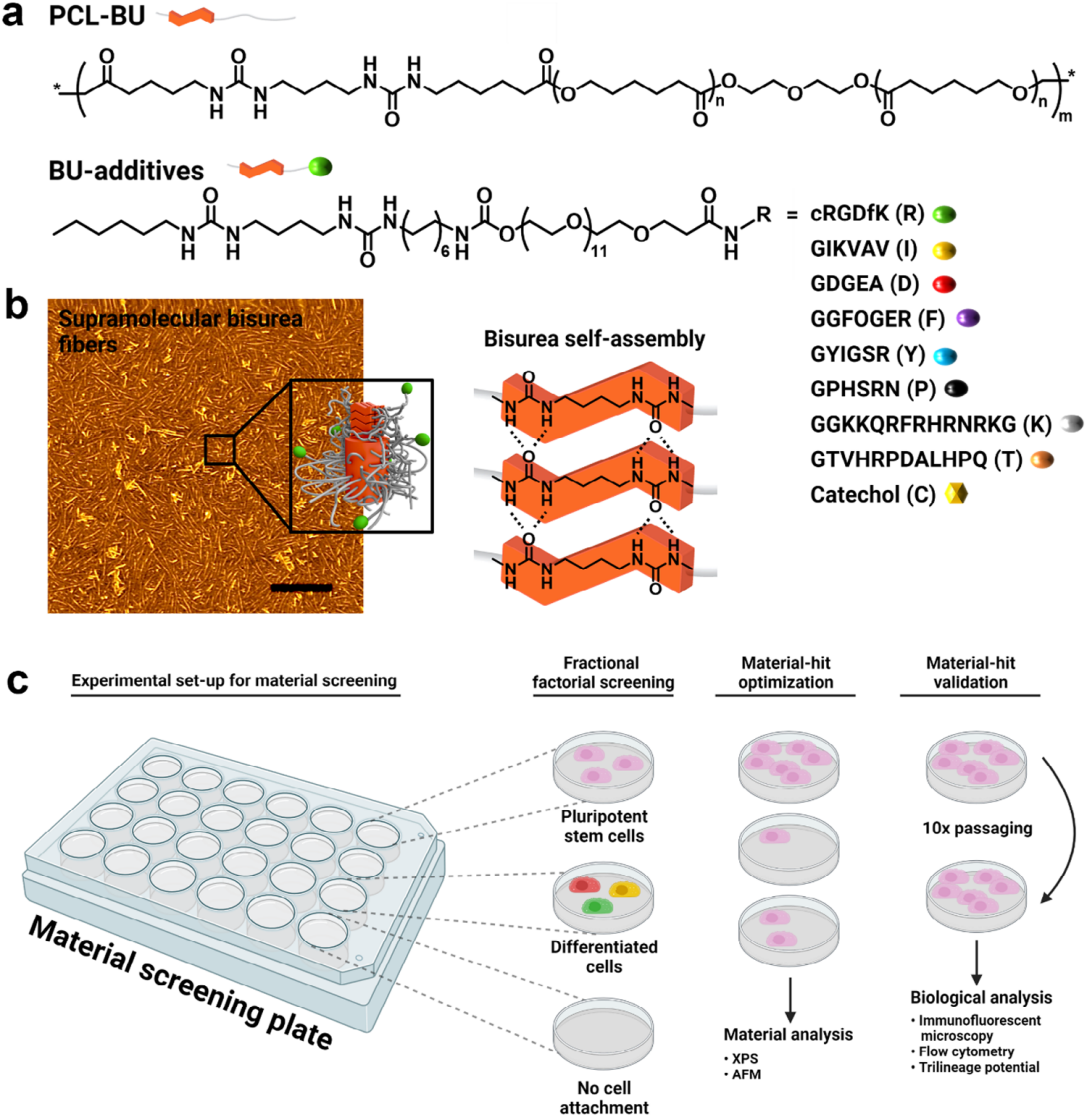
Schematic overview of the bisurea material library screening. a) Chemical structures of PCL‐BU and BU‐additives. The R‐group refers to the listed peptide sequences or catechol group, followed by a one‐letter code that is used as abbreviation. b) Atomic force phase micrograph of a PCL‐BU polymeric thin film depicting hard phase BU fiber‐like structures. To the right is a schematic representation of self‐assembly of BU moieties. Scale bar, 200 nm. c) A schematic overview of the bisurea materials screening and follow‐up experimental workflow.

Electrospinning is a material processing technique in which organic polymer solutions are exposed to an electric field resulting in deposition of a fibrous mesh on a rotating collector.^[^ ^28^, ^29^, ^30^ ^]^ Using this technique, it is possible to fabricate microfibers with a diameter ranging from 0.01–10 µm, which resemble the cylindrical geometric shape.^[^ ^31^, ^32^, ^33^, ^34^ ^]^ Supramolecular materials with bisurea (BU) motifs are eminently suited for material processing using the electrospinning technique.^[^ ^35^, ^36^, ^37^ ^]^ The segmented poly(ε‐caprolactone) bisurea polymer (PCL‐BU) (**Figure** **1**a) is a supramolecular material that has been processed with this technique into scaffolds used for diverse biomedical applications such as vascular grafts and heart valves.^[^ ^38^, ^39^, ^40^ ^]^ Furthermore, a wide variety of cell types have been shown to adhere to this material.^[^ ^37^, ^41^, ^42^, ^43^, ^44^ ^]^ The bisurea (BU) moieties present in this polymer self‐associate into ribbon‐like structures through bifurcated hydrogen bonds between urea groups, which are shielded by an adjacent alkyl spacer. ≈3–6 ribbon structures self‐assemble into nanofibers (Figure 1b).^[^ ^37^, ^45^, ^46^, ^47^ ^]^ The self‐associative behavior of the BU moieties allows for a modular approach in which various BU‐additives with complementary supramolecular moieties are mix‐and‐matched into the bulk material, without the need for additional chemical modification.^[^ ^35^, ^36^, ^42^, ^43^, ^44^, ^48^, ^49^, ^50^ ^]^ This modular approach is highly complementary to material screening approaches, which allows the discovery of supramolecular additive combinations that induce complex biological responses.^[^ ^44^ ^]^

Here, we used a fractional factorial screening of a biomaterial library containing BU‐additive combinations to identify supramolecular material formulations that induce hiPSC adhesion and maintenance of their pluripotent state. This statistical screening method enables limited screening conditions to provide information on which component has the largest effect on a read‐out, thereby reducing the total number of screening conditions required.^[^ ^51^ ^]^ A total of eight peptide additives were used: (1–2) cRGDfK^[^ ^52^ ^]^ and GPHSRN^[^ ^53^ ^]^ derived from fibronectin, (3–4) GIKVAV^[^ ^54^ ^]^ and GYIGSR^[^ ^55^ ^]^ derived from laminin, (5–6) GDGEA^[^ ^56^ ^]^ and GGFOGER^[^ ^57^ ^]^ derived from collagen type I, but lacking the flanking GPP‐repeat sequence that induces triple helix conformation, (7) GGKKQRFRHRNRKG^[^ ^58^, ^59^ ^]^ derived from vitronectin, and (8) GTVKHRPDALHPQ^[^ ^21^ ^]^ derived from a phage‐display study that improved stem cell adhesion. A ninth BU‐additive was used in the screening that contained a catechol group, which acts as an adhesive to improve protein anchoring to a material (Figure 1a).^[^ ^44^ ^]^ The BU‐additive combination that was selected based on the fractional factorial screening was further optimized by variation in additive concentration and validated by assessing the long‐term pluripotency of hiPSCs through consecutive passaging on the screening‐hit for a period of 35 days. After validation of the screening‐hit, the material was processed into microfibrous rafts that enabled hiPSC dynamic suspension culture without the undesired clumping behavior observed on microcarrier beads.

## 2 Results and Discussion

### 2.1 Material Screening for Stem Cell Adhesion and Maintenance of Pluripotent State

A material library with different BU‐additive combinations was set up using a fractional factorial design with a design resolution of IV, allowing the estimation of the effect that a single additive and a combination of two additives have. This method of screening has the benefit of only evaluating a limited number of material combinations as opposed to a full factorial screening, while still obtaining information on which material components influence the read‐out parameters the most. As such, a total of 32 different additive combinations (Table S1, Supporting Information) were processed into thin polymeric films on glass coverslips (Ø = 13 mm) via drop casting procedure. The BU‐additives were incorporated into the thin films at a concentration of 1 mol% relative to the repeating unit of the segmented PCL‐BU polymer. On these materials, hiPSCs were cultured for 3 days to screen which additives had the largest effect on hiPSC adhesion and pluripotency (Figure 1c). No hiPSCs were able to adhere to pristine PCL‐BU drop cast films or PCL‐BU with 1 mol% of a single BU‐additive. Exceptions were BU‐cRGDfK and BU‐catechol, respectively, which allowed adhesion of a small number of hiPSCs (Figure S1, Supporting Information). Furthermore, the introduction of the BU‐GFOGER additive into the material was unable to induce cell adhesion. The GFOGER sequence was included without the flanking GPP‐repeat sequence that induces triple‐helix formation and is required for integrin‐ligand interaction. Our rationale behind excluding this sequence was to deduce whether the resulting supramolecular assembly was able to compensate for the absence of the triple‐helix forming sequence. However, we observed that this hypothesis does not hold.

**Figure 2:**
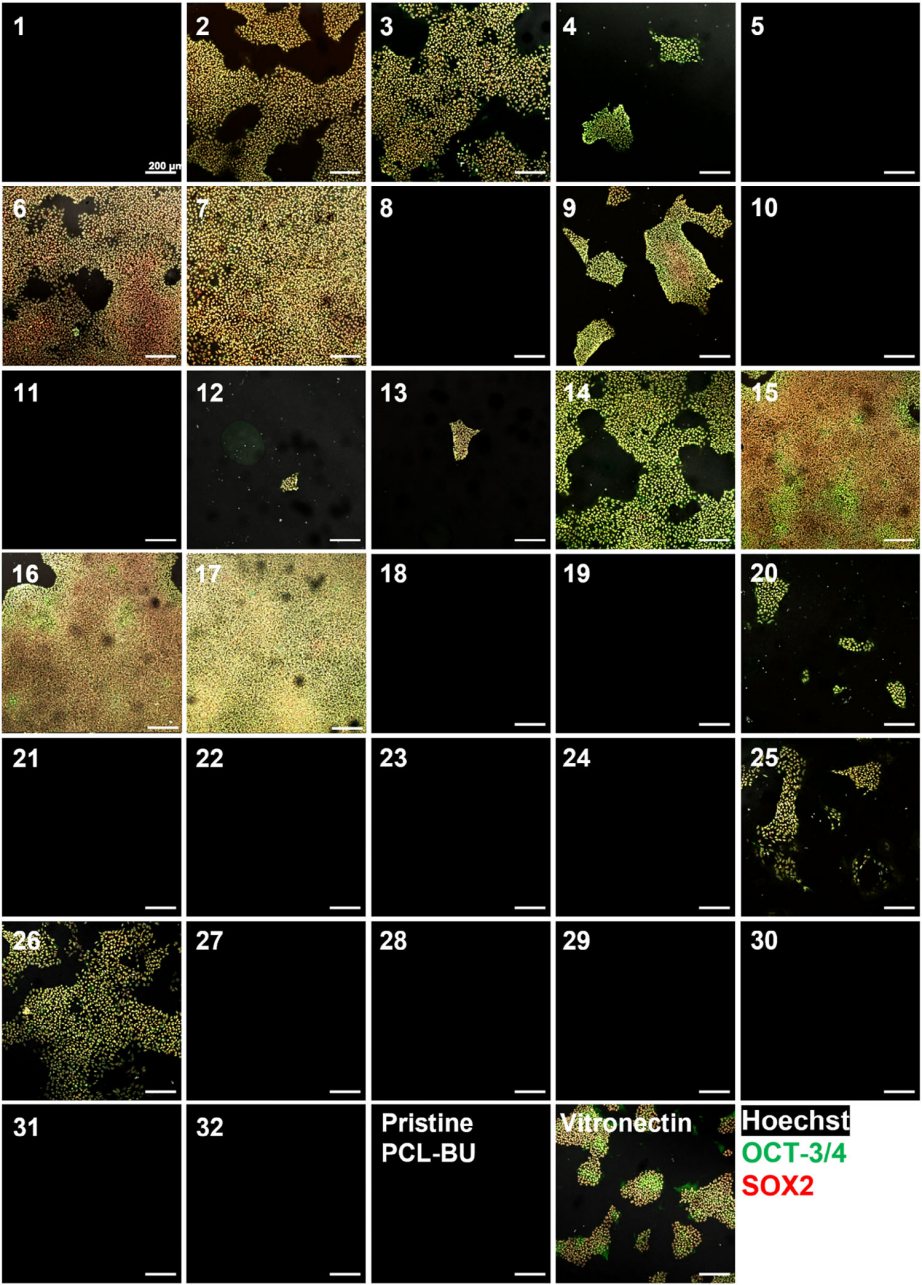
Representative immunofluorescence microscopy images of hiPSCs on bisurea materials derived from the fractional factorial screening library after 3 days of culture. Black images are BU‐based materials on which no hiPSCs were found after 3 days of culture. Scale bars, 200 µm.

The introduction of BU‐additive combinations into the material improved the cell‐material interactions. A total of 13 BU‐additive combinations were able to induce cell adhesion with large differences in both cell number and pluripotency percentage, respectively, between material conditions (**Figure** **2**). This demonstrated the synergistic effect of these BU‐additives on cell adhesion, proving the advantage of a modular material library. All materials that enabled hiPSC adhesion contained the BU‐cRGDfK additive, which indicates that this peptide sequence is required.

**Figure 3:**
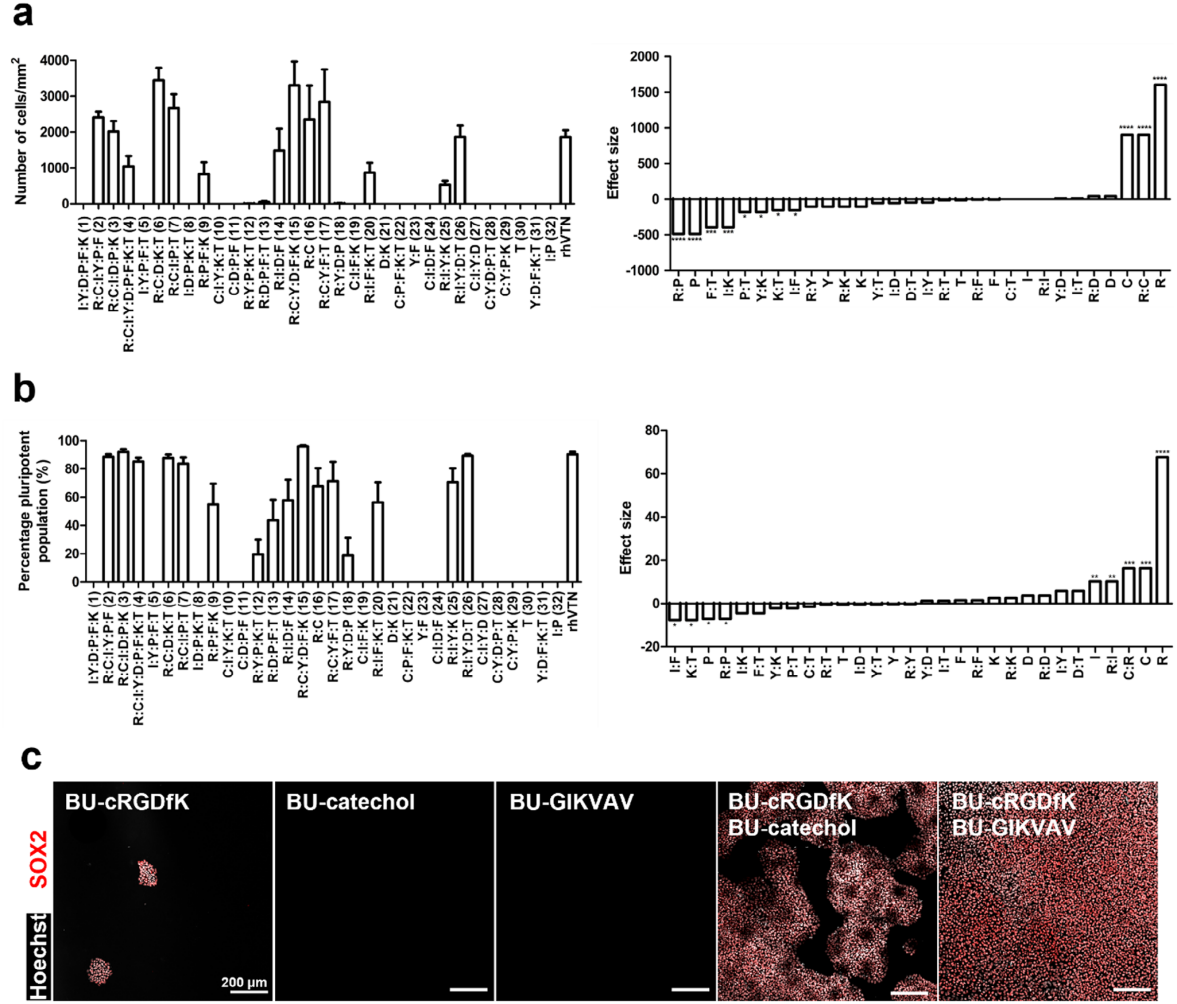
Fractional factorial screening output on hiPSC adhesion and pluripotency. a) The left graph shows the quantification of hiPSC count on bisurea materials after 3 days of culture. Data are represented as a mean ± s.e.m., N = 3. The right graph shows the effect size of single BU‐additives and two additive combinations. b) The left graph shows the quantification of hiPSC pluripotency on bisurea materials after 3 days of culture. Data are represented as a mean ± s.e.m., N = 3. The right graph shows the effect size of single BU‐additives and two additive combinations. c) Immunofluorescent microscopy images of hiPSCs on BU‐additive materials that had a significant effect size on pluripotency percentage. Scale bars, 200 µm. a,b) Statistical significance was attributed to values of *p* < 0.05 as determined by an effect normal plot. *p < 0.05, **p < 0.01, ***p < 0.001, ****p < 0.0001.

The number of hiPSCs after 3 days of culture on each material condition was determined by counting the nuclei, which were stained with Hoechst 33 342. Furthermore, the percentage of hiPSC population that remained pluripotent after 3 days of culture was quantified by immunostaining for OCT‐3/4 and SOX2. These results were used to determine the absolute effect size of single additives and two‐additive combinations on the total number of cells and pluripotency (**Figure** **3**b). Based on the fractional factorial screening, the BU‐cRGDfK additive, BU‐catechol, and a combination of the two had a significant positive effect on the total number hiPSCs. Furthermore, BU‐cRGDfK, BU‐catechol, and BU‐GIKVAV were single additives that had a significant positive effect on maintaining pluripotency of hiPSCs. A combination of BU‐cRGDfK and BU‐catechol, and BU‐cRGDfK and BU‐GIKVAV also had a positive effect on pluripotency.

The screening hits were processed into polymeric films, and hiPSCs were seeded on top and cultured for 3 days. Single BU‐additives were not or barely able to induce hiPSC adhesion after this culture period. Combining the BU‐additives increased the number of cells found on the drop cast films after 3 days of culture, with a combination of BU‐cRGDfK and BU‐GIKVAV demonstrating the highest number of cells with almost a complete monolayer after the culture period (Figure 3c). This was not expected based on the fractional factorial screening, which showed that this combination should have no effect on cell number per surface area. However, the positive effect that this combination has may be inhibited by other additive combinations that have a negative effect on hiPSC adhesion. Furthermore, it is not clear whether the increase in number of hiPSCs is caused by improved initial hiPSC adhesion, or that the cell‐material interaction promotes proliferation.

### 2.2 Screening‐Hit Optimization

**Figure 4:**
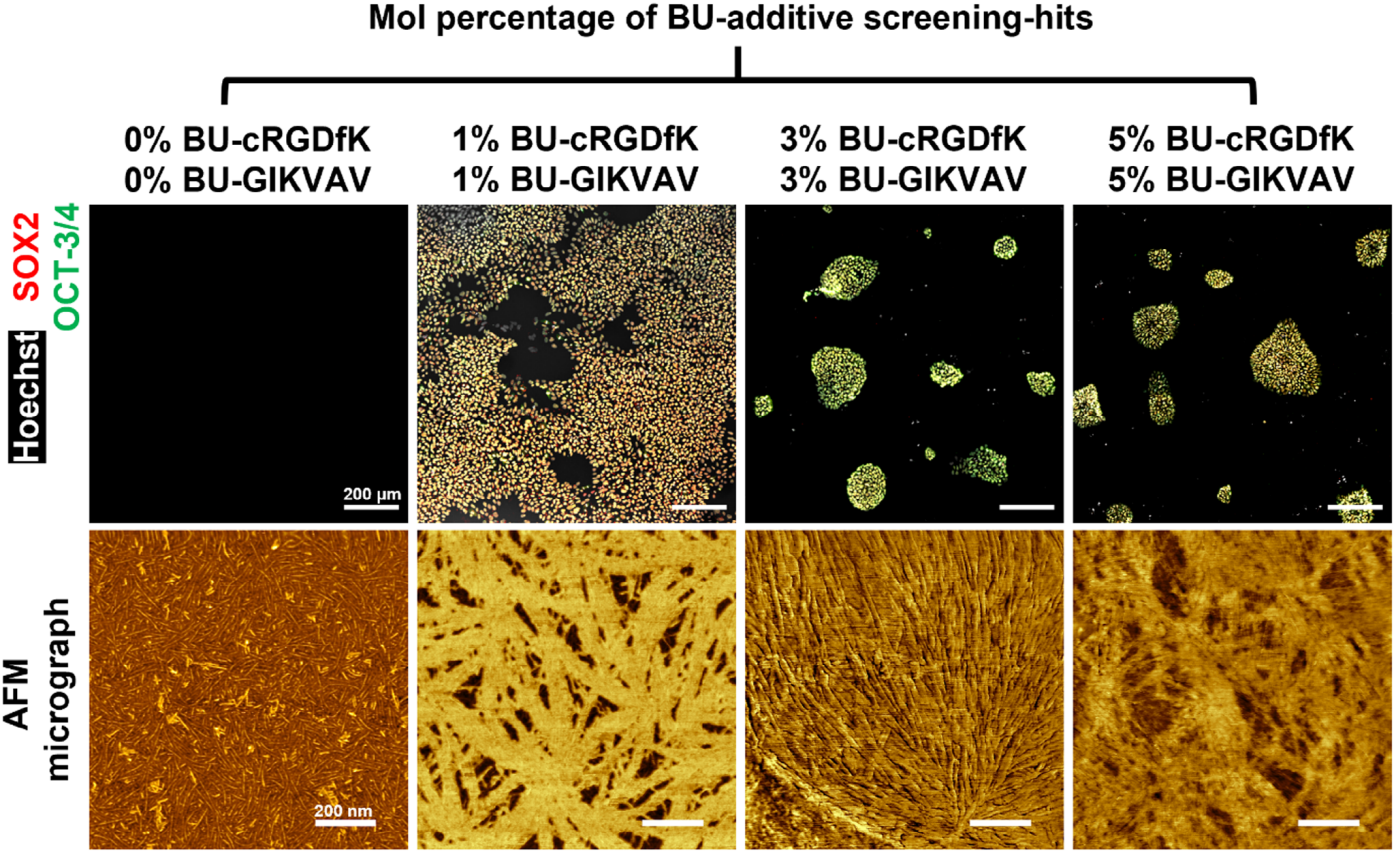
Screening‐hit optimization by variation of additive mol percentage. Top row shows immunofluorescent images of hiPSCs after a 3‐day culture period on bisurea materials with variations in additive concentration of the screening hit. Scale bars, 200 µm. Bottom row shows atomic force phase micrographs of PCL‐BU polymeric thin films with variations in additive concentration of the screening hits. Scale bars, 200 nm.

The screening‐hit optimization was continued with the BU‐cRGDfK and BU‐GIKVAV additive combination by varying their concentration (0, 1, 3, or 5 mol%, respectively) in the material. We reasoned that increasing their concentration results in a higher ligand density for integrins on the hiPSC surface, which potentially promoted adhesion capacity. Contrary to our expectations, increasing the concentration of each additive to 3 or 5 mol% decreased the ability of cells to adhere to the material (**Figure** **4**). Atomic force microscopy (AFM) phase images show nanofiber formation in the PCL‐BU films without additives. Upon introduction of 1 mol% of each of the two additives, a change in the surface architecture is observed, with distinct crystalline features (Figure. 4). Quantification of the atom composition at the material surface using XPS measurements provides an insight into the ratio of three components at the material surface. The XPS measurement shows an increase in nitrogen and oxygen content upon introduction of 1 mol% BU‐additives as compared to the pristine PCL‐BU material (**Table** **1**). This is an indication that the change in material surface architecture, as observed in the AFM micrographs, contains the BU‐additives, which phase separated from the bulk PCL‐BU polymer. Increasing the respective additive concentrations further to 3 or 5 mol% increased the nitrogen and oxygen content at the surface even more. The increase of nitrogen and oxygen at the surface was accompanied by more crystalline domains forming at the material surface as seen by AFM, which completely covers the surface (Figure 4). This suggests that increasing the additive concentrations to >3 mol% decreases the additive incorporation in the bulk material. It is possible that this makes the BU‐additives located at the surface more susceptible to leaking out of the material. If that indeed is the case, then the cells that adhere to these leakage‐susceptible additives are also prone to detach from the surface. Together this data demonstrates that a combination of BU‐cRGDfK and BU‐GIKVAV at a concentration of 1 mol%, respectively, results in the optimal adhesion of hiPSCs while maintaining their pluripotency. Henceforth, we refer to this additive combination as the “material‐hit” based on the fractional factorial screening and subsequent hit‐optimization.

**Table 1:** Atom composition at the surface of bisurea materials with variations in additive concentration based on X‐ray photoelectron spectroscopy measurements.

Mol composition [%]			Atom composition [at. %]			Ratio [C/N]
PCL‐BU	BU‐cRGDfK	BU‐GIKVAV	Carbon	Nitrogen	Oxygen	
100	0	0	84.5	1.7	13.8	51.2
98	1	1	70.7	10.0	19.3	7.1
94	3	3	68.2	12.0	19.8	5.7
90	5	5	66.1	13.3	20.6	5.0

### 2.3 Long‐Term Maintenance of Pluripotency by Material‐Hit

**Figure 5:**
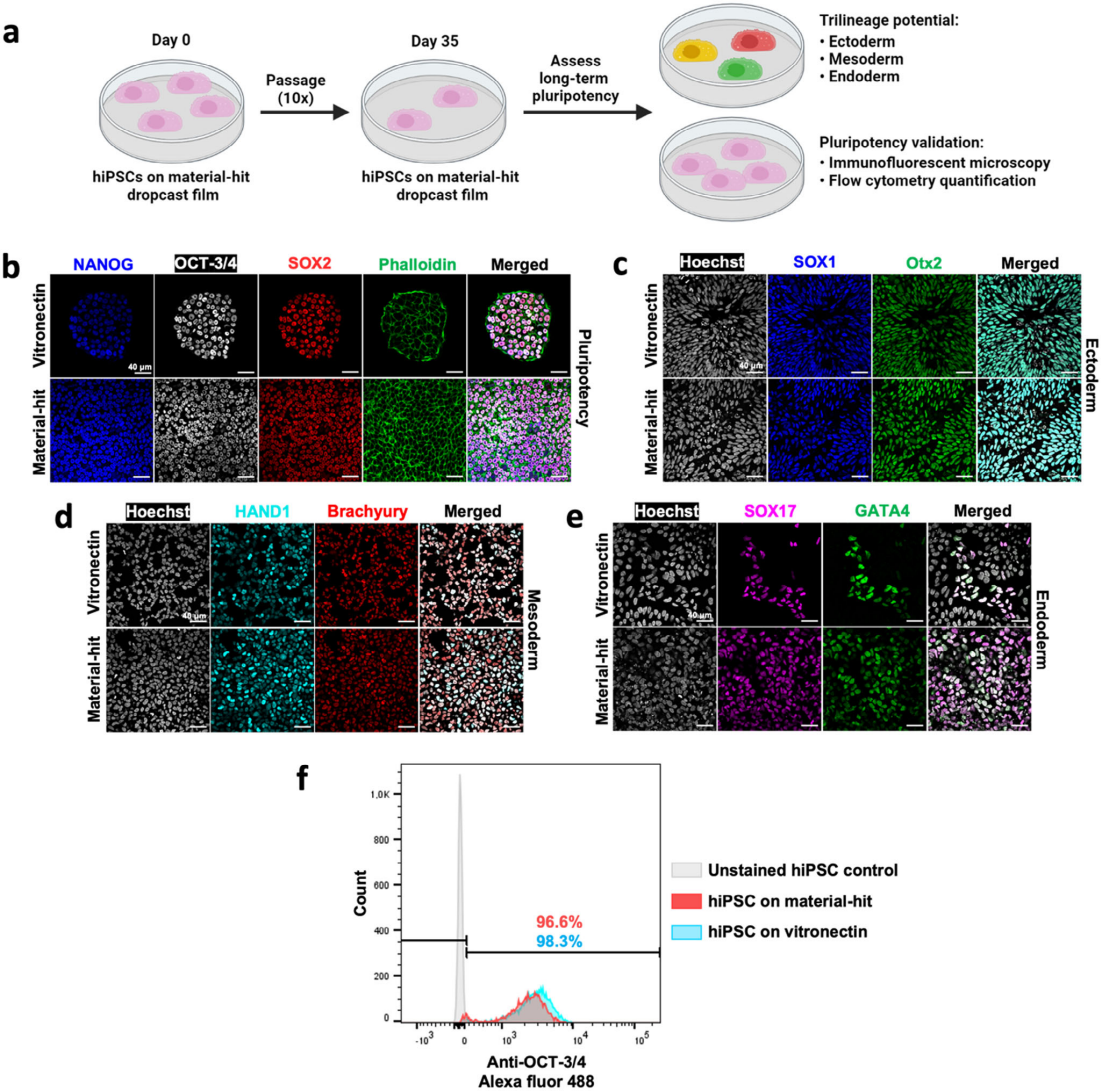
Material‐hit validation for hiPSC pluripotency after 35 days. a) A Schematic overview of the experimental workflow to assess long‐term pluripotency of hiPSCs on the bisurea material‐hit. b) Immunofluorescence microscopy images of hiPSCs after 4‐day culture period, staining transcription factors NANOG, OCT‐3/4, and SOX2. c) Immunofluorescence images of ectodermal cells after a 7‐day differentiation period, staining SOX1 and Otx2. d) Immunofluorescence images of mesodermal cells after a 5‐day differentiation period, staining HAND1 and Brachyury. e) Immunofluorescence images of endodermal cells after a 5‐day differentiation period, staining SOX17 and GATA4. b–e) Scale bars, 40 µm. f) Plot shows flow cytometry quantification of OCT‐3/4^+^ cells.

The hiPSCs require prolonged expansion involving multiple passaging steps to acquire a sufficient number of stem cells for regenerative therapeutic applications. To assess whether the chemical composition of the material‐hit supports prolonged hiPSC expansion, ten consecutive passages (i.e., 35‐day culture period) on drop casted films of the material‐hit were performed (**Figure** **5**a). In parallel, hiPSCs were also passaged on glass coverslips coated with recombinant vitronectin human protein as comparison. It was noted that it was more difficult to enzymatically detach cells from the material‐hit surface compared to the vitronectin‐coating using TrypLE select enzyme, with cells requiring 1.5× longer incubation time with the enzyme solution to be detached. After ten passages, the long‐term pluripotent state of the cells was assessed through immunostaining of OCT‐3/4, SOX2, and NANOG. Cells on both the material‐hit and vitronectin‐coating maintained expression of these transcription factors associated with pluripotency (Figure 5b). The percentage of pluripotent cells was quantified using flow cytometric analysis of OCT‐3/4^+^ pluripotent population, with both material conditions enabling pluripotency maintenance of >95% of the population (Figure 5f). The amount of OCT‐3/4^+^ cells was slightly higher on the vitronectin‐coated surfaces (98.3%) compared to the material‐hit surface (96.6%). However, we assume this difference to be negligible. Furthermore, as a potency assay, we investigated the trilineage potential of hiPSCs cultured for >1 month on both surfaces by differentiation to the three germ layers (i.e., ectoderm, mesoderm, and endoderm). The trilineage assay is considered proof for full pluripotent lineage potential. To this end, after ten passages, hiPSCs were differentiated toward ectoderm (SOX1^+^ and Otx2^+^), mesoderm (HAND1^+^ and Brachyury^+^), and endoderm (SOX17^+^ and GATA4^+^) on a drop cast film of the material‐hit or on the vitronectin‐coating, respectively. The hiPSCs cultured and differentiated on the material‐hit demonstrated full trilineage potential through differentiation to all germ layers (Figure 5c–e). Together, these results confirmed the ability of the material‐hit to maintain long‐term pluripotent potential in hiPSCs to a similar degree as vitronectin.

### 2.4 Electrospinning Microfibrous Rafts for Dynamic Suspension Culture

**Figure 6:**
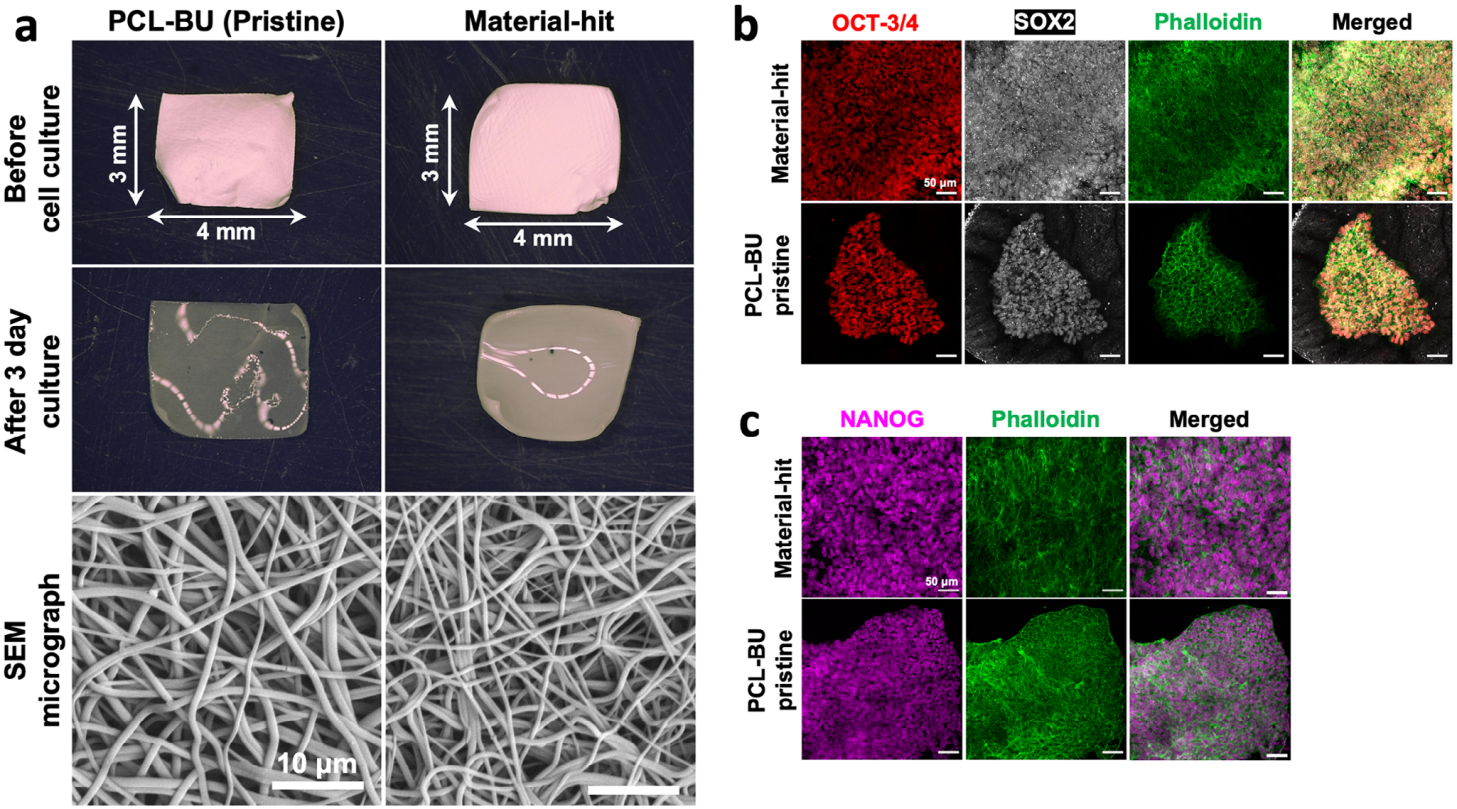
Bisurea‐based carrier platforms for expansion of hiPSCs. a) Top‐row depicts digital microscopy images of electrospun meshes after being cut into carrier platforms, middle row shows these carrier platforms after a 3‐day culture period. The bottom row shows SEM micrographs of the electrospun meshes. Scale bars, 10 µm. b–c) Immunofluorescence confocal microscopy images of hiPSCs after a 3‐day suspension culture period on carrier platform. Scale bars, 50 µm.

In order to increase the scale of hiPSC expansion, it is important to move from a standard 2D culture toward a dynamic 3D suspension culture to increase the surface area accessible to cells. Bead‐shaped microcarriers are already commercially available and used for 3D suspension culture of hiPSCs. However, beads have the lowest surface‐to‐volume of all geometric shapes and lead to clumping, which makes the suspension culture uncontrolled. Furthermore, the effect of curvature on hiPSC differentiation needs to be considered and evaluated, as it may initiate undirected differentiation or affect germ‐layer specification.^[^ ^60^, ^61^, ^62^ ^]^ An easy‐producible geometric shape with a higher surface‐to‐volume ratio is a cylinder or fiber structure, which can be produced by electrospinning. Previous work has demonstrated that electrospun PCL scaffolds with‐ or without peptides allow hiPSC adhesion.^[^ ^63^, ^64^ ^]^ Furthermore, sectioning the electrospun scaffolds into smaller pieces allows for the fabrication of microfibrous rafts, which allow for planar hiPSC culture in dynamic suspension culture. To produce these microfibrous rafts, polymer solutions of PCL‐BU and the material‐hit, at a concentration of 130 mg mL^−1^, were electrospun into meshes. The PCL‐BU mesh without BU‐additives contained fibers with a radius of 383 ± 21 nm, and the fibers in the electrospun mesh from the material‐hit (i.e., PCL‐BU with BU‐additives) had a radius of 292 ± 14 nm (**Figure** **6**a). The radius of these fibers is too small for an improvement in terms of surface‐to‐volume ratio compared to bead‐shaped microcarriers, due to the cells being larger (≈10–15 µm on their longest axis) than the fibers. We anticipate that the fiber radius requires at least a ten‐fold increase for multiple hiPSCs to adhere to a single electrospun fiber. However, this does not affect the raft cell carrier configuration, as these fiber diameters still allow a planar culture of the hiPSCs in a dynamic suspension culture set‐up. As such, the bisurea polymeric meshes were cut into microfibrous rafts of ≈ 3 × 4 mm, and twenty of these were placed in a non‐coated 24‐well plate on an orbital shaker. This amounts to a total of 4.8 cm^2^ per well, as compared to the 1.9 cm^2^ of a 24‐well plate. This means that the hiPSCs that were cultured in this dynamic suspension culture set‐up under shaking conditions had access to >2.5× more surface area compared to when cultured on the well‐bottom. It should be noted that twenty of these carrier platforms occupied only a small fraction of the total volume in each well, which means that the total possible cell culture area may easily be increased.

The hiPSCs were seeded in a 24‐well plate containing microfibrous rafts on an orbital shaker to assess the capacity of the rafts in supporting of hiPSC expansion in a dynamic suspension culture set‐up. After a 3‐day culture period, the hiPSCs adhered to both microfibrous rafts with‐ and without BU‐additives. In both conditions, the cells formed either large colonies or covered the entire material surface on both sides of the raft. Interestingly, these results are contrary to what was observed for drop casted bisurea polymer films, in which incorporation of BU‐cRGDfK into the PCL‐BU drop casted films was required for hiPSC adhesion. The surface roughness that is introduced to the material through electrospinning by the microfibers is most likely a beneficial cue for cytoskeletal rearrangement in hiPSCs, which in turn improves cell adhesion.^[^ ^65^ ^]^

On both raft materials, hiPSCs maintained a pluripotent state during the 3 days in dynamic suspension culture, evident by expression of NANOG, OCT‐3/4, and SOX2 (Figure 6b,c). Furthermore, the planar configuration of the raft‐shaped carriers avoided clumping, which is observed in bead‐shaped microcarriers. This demonstrates that other carrier platform configurations, besides microbeads, are able to support the expansion of pluripotent stem cells in a dynamic suspension culture set‐up while maintaining their pluripotent state.

## 3 Conclusion

We reported a supramolecular material library that was screened in a fractional factorial manner to identify compounds capable of maintaining long‐term pluripotency of stem cells. The compounds that were identified as screening‐hits were refined into a material suitable for pluripotent stem cell expansion. This fully synthetic material‐hit was processed into a raft‐like carrier platform with fiber‐shaped microstructures through electrospinning. Application of these microfibrous rafts allowed for adherent pluripotent stem cell culture in a dynamic suspension culture set‐up, which increased the surface area accessible for cell expansion compared to static adherent culture in well plates. The pluripotent stem cells were able to adhere to PCL‐BU carriers with, ‐and without the BU‐additives found, which was contrary to what was observed in the material screening. This difference is attributed to the surface roughness, which indicates that this material parameter is enough to induce a hiPSC‐material interaction. Most importantly, this work demonstrates that the classic bead‐shaped microcarriers are not the ideal carrier platform. Additional investigation is required in elucidating the advantages of different geometrical carrier shapes, their microstructural composition, and chemical composition. Based on this work, we expect that optimization of these three parameters enables the formulation of microcarriers that enable dynamic suspension culture in an efficient and reproducible manner that conserves the pluripotent phenotype.

## 4 Experimental Section

### Synthesis of BU‐Additives

The BU‐cRGDfK, BU‐GGFOGER, BU‐GYIGSR, BU‐GPHSRN, BU‐GDGEA, and BU‐catechol additive were synthesized as previously reported.^[^ ^43^, ^44^ ^]^

### Bisurea Material Screening Library Preparation

A polymer solution (7.4 mm; per segmented unit) was produced by dissolving PCL‐BU (Mn = 2700 g mol^−1^; per segmented unit, SyMO‐Chem) in hexafluoroisopropanol (HFIP; Fluorochem) at a concentration of 20 mg mL^−1^ under stirring conditions at room temperature. BU‐additive solutions (7.4 mm) were prepared by dissolving BU‐additives in freeze‐dried powder form in 100 µL HFIP, respectively. Different BU‐additive combinations were mixed with the PCL‐BU solution in 1.5 mL glass vials. Afterward, 50 µL of bisurea mixture was drop casted on top of a glass coverslip (Ø = 13 mm) via pipetting. The drop casted polymeric bisurea films were placed in vacuo overnight to ensure evaporation of HFIP. Finally, the materials were UV‐sterilized for 15 min before used for cell culture.

### Microfibrous Raft Fabrication

Bisurea polymer solutions were produced by dissolving PCL‐BU in HFIP at a concentration of 130 mg mL^−1^ with or without 1 mol% BU‐cRGDfK and BU‐GIKVAV, respectively. The solutions were electrospun on an EC‐CLI (IME Technologies), and a polymeric mesh was collected on a cylindrical collector (Ø = 28 mm) wrapped in aluminum foil while rotating at a speed of 500 rpm. The polymer solution was fed through a nozzle (Ø = 1.0–0.8 mm) with a rate of 30 µL min^−1^. The distance between the tip of the nozzle and the collector was 13 cm. A voltage difference of 25 kV was applied to enable spinning of ≈600 µL. The chamber temperature was set to 23 °C and the relative humidity was 30%. The electrospun meshes were placed in vacuo overnight to remove residual HFIP. Afterward, the meshes were cut into shapes of ≈ 3 × 4 mm.

### Standard Culture of hiPSCs

hiPSCs (LUMC0072iCTRL04) were routinely cultured on 6‐well culture plates coated with 5 µg mL^−1^ vitronectin recombinant human protein, truncated (Thermofisher Scientific) in E8 medium (Thermofisher Scientific). Cells were passaged as single cells using TrypLE Select (Thermofisher Scientific) at a density of 20.000 cells cm^−2^, with RevitaCell supplement (Thermofisher Scientific) added in the first 18–24 h. The E8 medium was replaced every 1–2 days. Cells were routinely checked for mycoplasma contamination.

### Dynamic Suspension Culture of hiPSCs

Microfibrous rafts were UV‐sterilized on both sides for 15 min. Afterward, the rafts were transferred to a 24‐well plate and 1 mL of RevitaCell supplemented E8 medium was added to the rafts, and the rafts were submerged using a pipette tip. The rafts were pre‐wetted by incubation in the supplemented medium for 24 h at 5% CO_2_ and 37 °C. The next day, hiPSCs (LUMC0072iCTRL04) were passaged as single cells using TrypLE Select (Thermofisher Scientific) and 100.000 cells were seeded per well containing the microfibrous rafts. After seeding, the well plate containing the rafts was placed on an orbital shaker (70 rpm) in an incubator set at 5% CO_2_ and 37 °C. After 24 h, the RevitaCell supplemented E8 medium was replaced by 1 mL of non‐supplemented E8 medium. The dynamic suspension culture was stopped on day 3 of culture.

### Differentiation of hiPSCs to Germ Layers

On day ‐1, hiPSCs were seeded on glass coverslips that were coated with recombinant vitronectin human protein or a drop casted film of PCL‐BU with 1 mol% BU‐cRGDfK and 1 mol% BU‐GIKVAV. The hiPSCs were seeded at a density of 200 000 cells cm^−2^ for ectoderm and endoderm differentiation, and 50 000 cells cm^−2^ for mesoderm differentiation. The cells were maintained in E8 medium supplemented with RevitaCell supplement for 24 h. On day 0, medium was switched to STEMdiff trilineage ectoderm medium, STEMdiff trilineage mesoderm medium, or STEMdiff trilineage endoderm medium. On day 5, differentiation toward mesoderm and endoderm was completed and the samples were fixated. On day 7, differentiation toward ectoderm was completed and the samples were fixated. The differentiation medium was changed daily.

### Immunohistochemistry

Samples were washed 2× with sterile PBS solution and fixated in 4% formaldehyde solution for 10 min at room temperature. Samples were washed 2x with PBS solution after fixation and incubated with 10% donkey serum + 0.3% Triton X‐100 in PBS solution for 30 min at room temperature. After blocking and permeabilization, samples were incubated with primary antibodies for 3 h at room temperature and incubated with secondary fluorescent‐conjugated antibodies (1:400 dilution) for 2 h at room temperature. Afterward, samples were stained with NucBlue Live ReadyProbes Reagent (Hoechst 33 342) (Thermofisher Scientific) for 10 min and washed three more times with PBS solution. All liquid was aspirated, and samples were mounted on glass coverslips (24 × 60 mm) in Mowiol. Mounted samples were imaged using a Leica TCS SP8 X confocal microscope (Leica Microsystems). Primary antibodies used in this study are listed in Table S2 (Supplementary Information).

### Flow Cytometry

After ten passages on the material‐hit or vitronectin coating, hiPSCs were enzymatically detached from the surface using 0.5 mL TrypLE, and 200 000 cells were transferred to 1.5 mL Eppendorf tube. The resulting cell suspension was diluted with 1 mL ice‐cold 1% bovine serum albumin (BSA) (Sigma‐Aldrich) solution in PBS and centrifuged for 3 min at 300 g. The supernatant was aspirated, and cells were resuspended in 1 mL BD Pharmingen TF Fix/Perm Buffer (BD Biosciences) at 4 °C. After 50 min, the fixated and permeabilized cells were centrifuged for 3 min at 300 g. The supernatant was aspirated, and fixated cells were resuspended in 1 mL BD Pharmingen TF Perm/Wash Buffer (BD Biosciences) (3×). Cells were stained with 100 µL anti‐OCT‐3/4 antibody solution (Santa Cruz Biotechnology) (1:100 dilution) at 4 °C. After 40 min, the cells were centrifuged for 3 min at 300 g. The supernatant was aspirated, and fixated cells were resuspended in 1 mL BD Pharmingen TF Perm/Wash Buffer (3×). Cells stained with anti‐OCT‐3/4 antibody were incubated with 100 µL anti‐mouse‐Alexa488 conjugated antibody (Jackson ImmunoResearch) at 4 °C. After 40 min, the cells were centrifuged for 3 min at 300 g. The supernatant was aspirated, and fixated cells were resuspended in 1 mL BD Pharmingen TF Perm/Wash Buffer (3×). The stained cells were resuspended in 1% BSA solution and analyzed on a BD FACS Canto II Flow Cytometer.

### Atomic Force Microscopy

AFM measurements were performed on a Bruker Dimension FastScan Bio Icon AFM in tapping mode using silicon cantilever tips (PPP‐NHCR, Nanosensors, 204–497 kHz, 10–130 N m^−1^). Scan areas were 500×500 nm^2^. Images were processed using Gwyddion software (version 2.52).

### Scanning Electron Microscopy

Electrospun meshes were visualized using the Phemon ProX desktop scanning electron microscope (SEM). The samples were taped onto aluminum stubs using double‐sided carbon tape and sputter coated with a thin layer of gold (40 mA, 30 s). Images were obtained at an electron energy of 15 kV and intensity setting “map”.

### X‐Ray Photoelectron Spectroscopy

XPS spectra were obtained using a Thermo Scientific K‐Alpha spectrometer equipped with a 180° double‐focusing hemispherical analyzer with a 128‐channel detector that uses an aluminum anode (Al Kα, 1486.7 eV, 72 W) and monochromatic, small‐spot X‐ray source. The survey scans used a pass energy of 200 eV and the atomic region scans 50 eV. The atom composition was quantified from the spectra using CasaXPS software (version 2.3.23).

### Screening Analysis of Microscopy Images

Acquired immunofluorescent microscopy images were analyzed using CellProfiler 4.1.3, applying custom‐made pipelines. The images were imported and converted to grayscale. The cell number mm^−2^ was determined using the total nuclei count per sample as captured by the Otsu global thresholding method applied on the DAPI image channel. The lower threshold bound was set at 0.2 to eliminate mis‐segmentation from background fluorescence. OCT‐3/4+ and SOX2+ cells were captured by the Otsu global thresholding method on the OCT‐3/4 and SOX2 channels, respectively. Here, the lower threshold bound was set at 0.15 to eliminate mis‐segmentation from background fluorescence. For all channels, objects were distinguished by intensity. The percentage pluripotent population was calculated by dividing the OCT‐3/4+ count by the total nuclei count. The same method was applied for determining the percentage of SOX2+ cells (Figure S2, Supplementary Information).

### Statistics

A fractional factorial design was used to define the initial screening conditions of the BU‐additive library. Design and analysis of the experiments was done using R (v3.4.4, R Foundation) with Rcmdr.DoE plugin (v0.12‐3, Ulrike Groemping). A 2‐level fractional factorial design with 9 factors for BU‐additives was selected with 32 unique conditions (Table S1). This resulted in a fractional factoring screening with design resolution IV. Main effects and two‐factor interactions were calculated according to standard fractional factorial analysis by R and the Rcmdr.DoE plugin. Statistical differences between conditions were tested with ANOVA analysis of variance followed by Tukey's post hoc test. Significance was assumed α ≤ 0.05. Different significance levels (P values) are indicated in each figure with asterisks: *p < 0.05, **p < 0.01, ***p < 0.001, and ****p < 0.0001.

## Conflict of Interest

The authors declare no conflict of interest.

## Supporting information



Supporting Information

## Data Availability

The data that support the findings of this study are available from the corresponding author upon reasonable request.
