# Supplementary material for: Supramolecular Additive Screening to Engineer Microfibrous Rafts for Expansion of Pluripotent Stem Cells in Dynamic Suspension
Source: Adv Healthc Mater. 2025 Mar 10;14(11):2404186. doi: 10.1002/adhm.202404186 (PMC12023819; doi:10.1002/adhm.202404186)
Supplement: Supplementary file 1 — Supporting Information [file ADHM-14-0-s001.docx]

Supporting Information

Supramolecular Additive Screening to Engineer Microfibrous Rafts for Expansion of Pluripotent Stem Cells in Dynamic Suspension

Johnick F. van Sprang, Jasper G.M. Aarts, Boris Arts, Joyce E.P. Brouns, Muhabbat I. Komil, Paul A.A. Bartels, and Patricia Y.W. Dankers


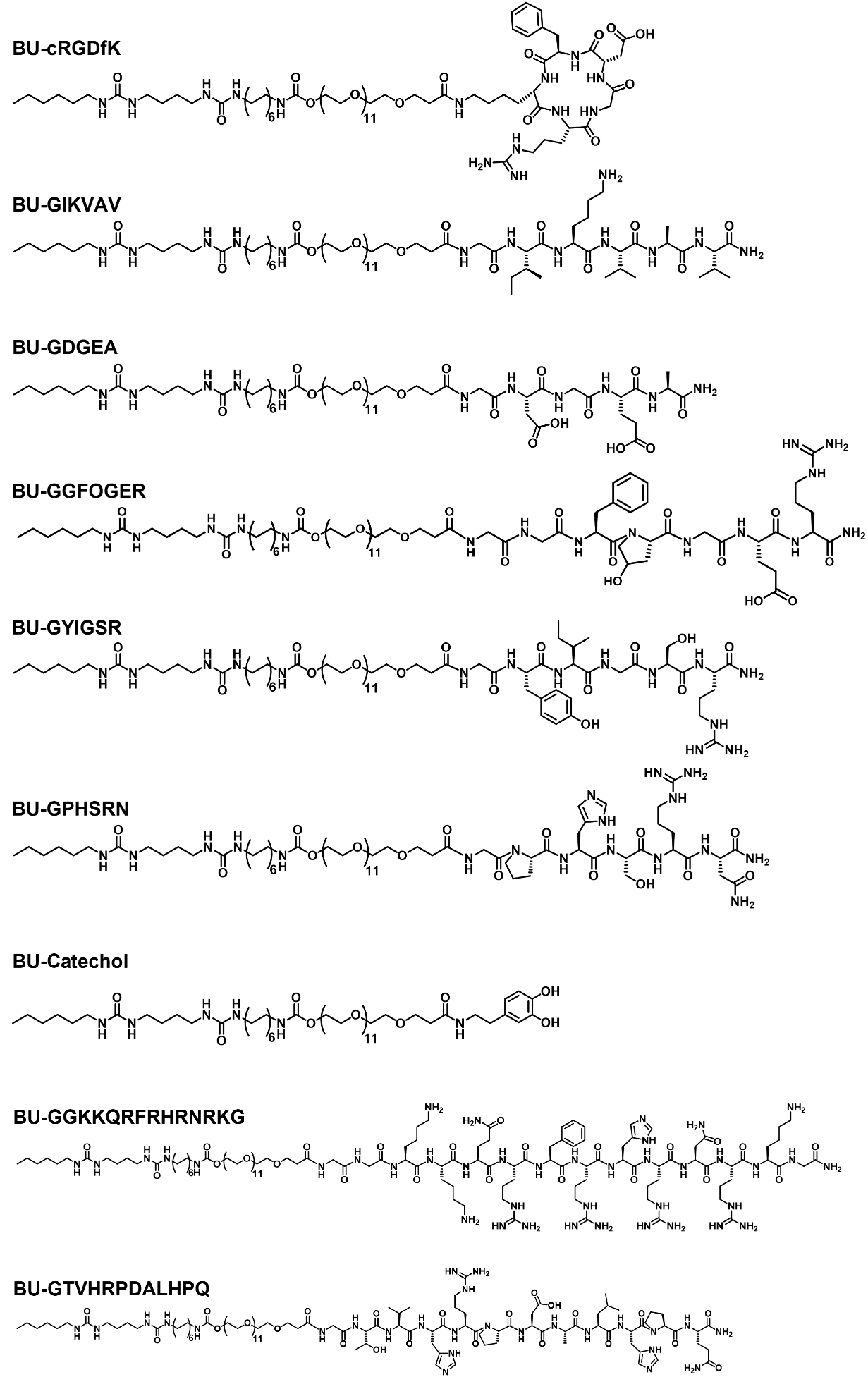


**Scheme S1.** **Chemical structures of BU-additives**.

**
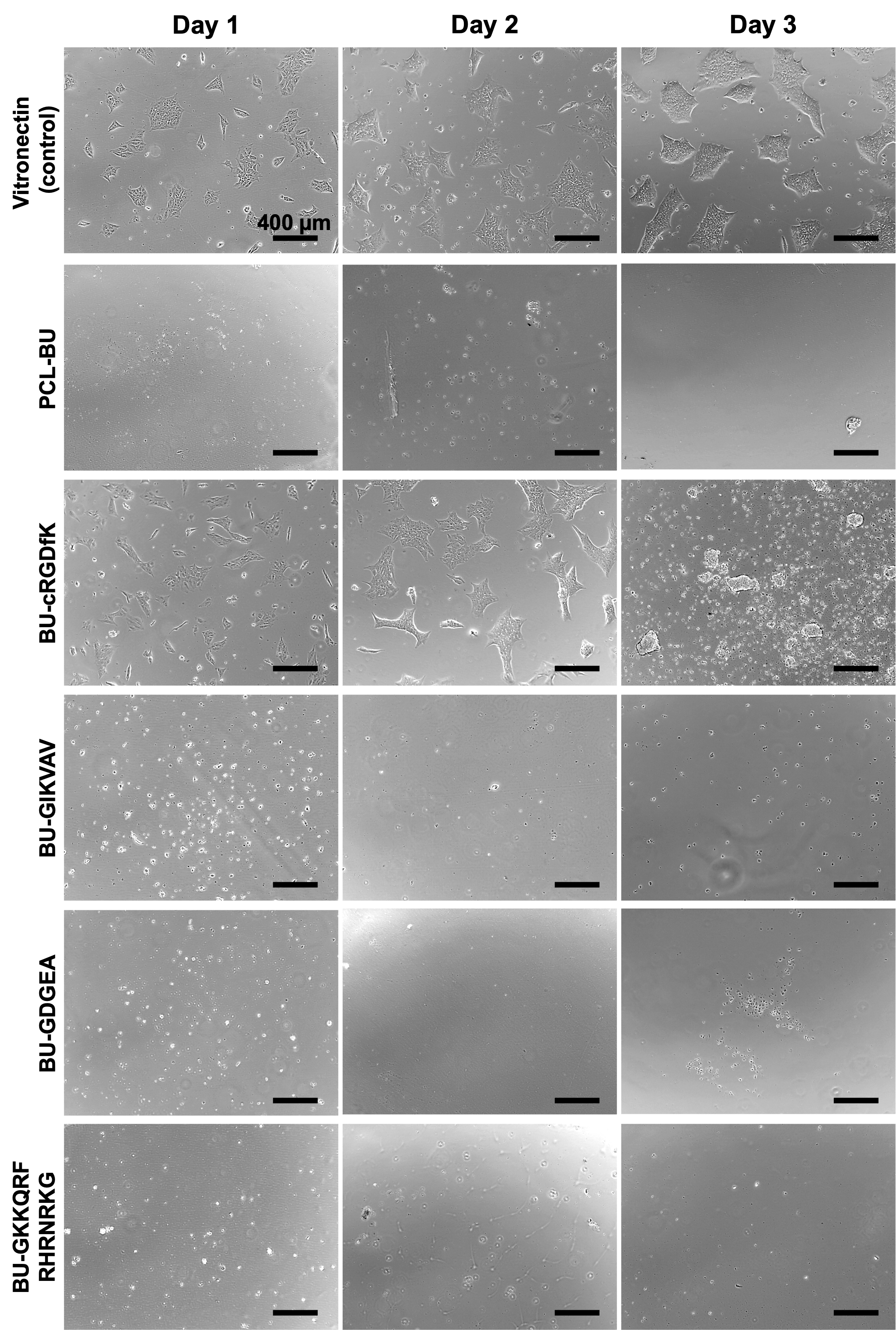
**


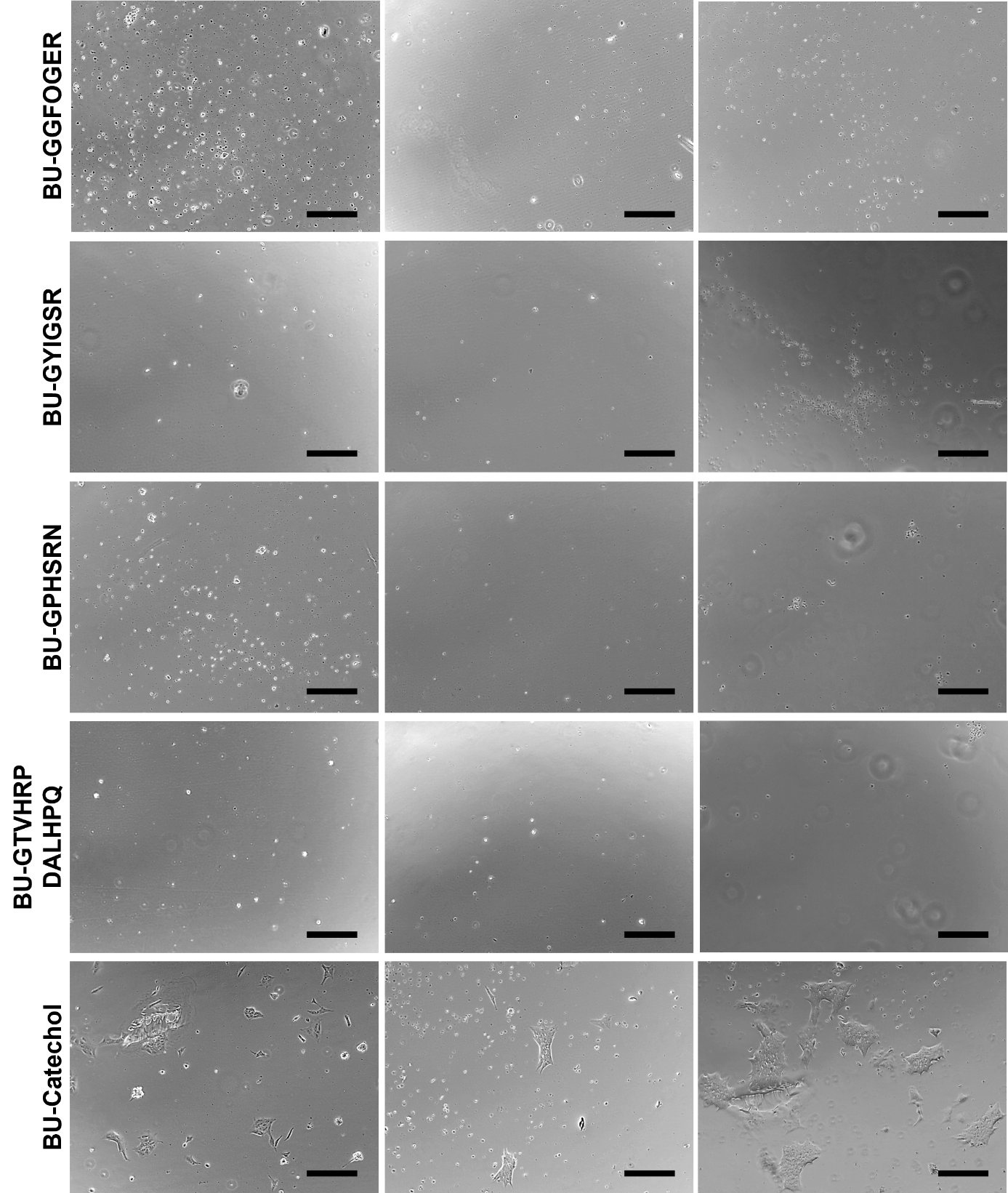


**Figure S1. hiPSCs on drop casted films with a single BU-additive.** Phase-contrast microscopy images show hiPSCs on drop casted PCL-BU films with each BU-additive, respectively, at a concentration of 1 mol%. Images show cells (or no cells) on day 1 – 3.


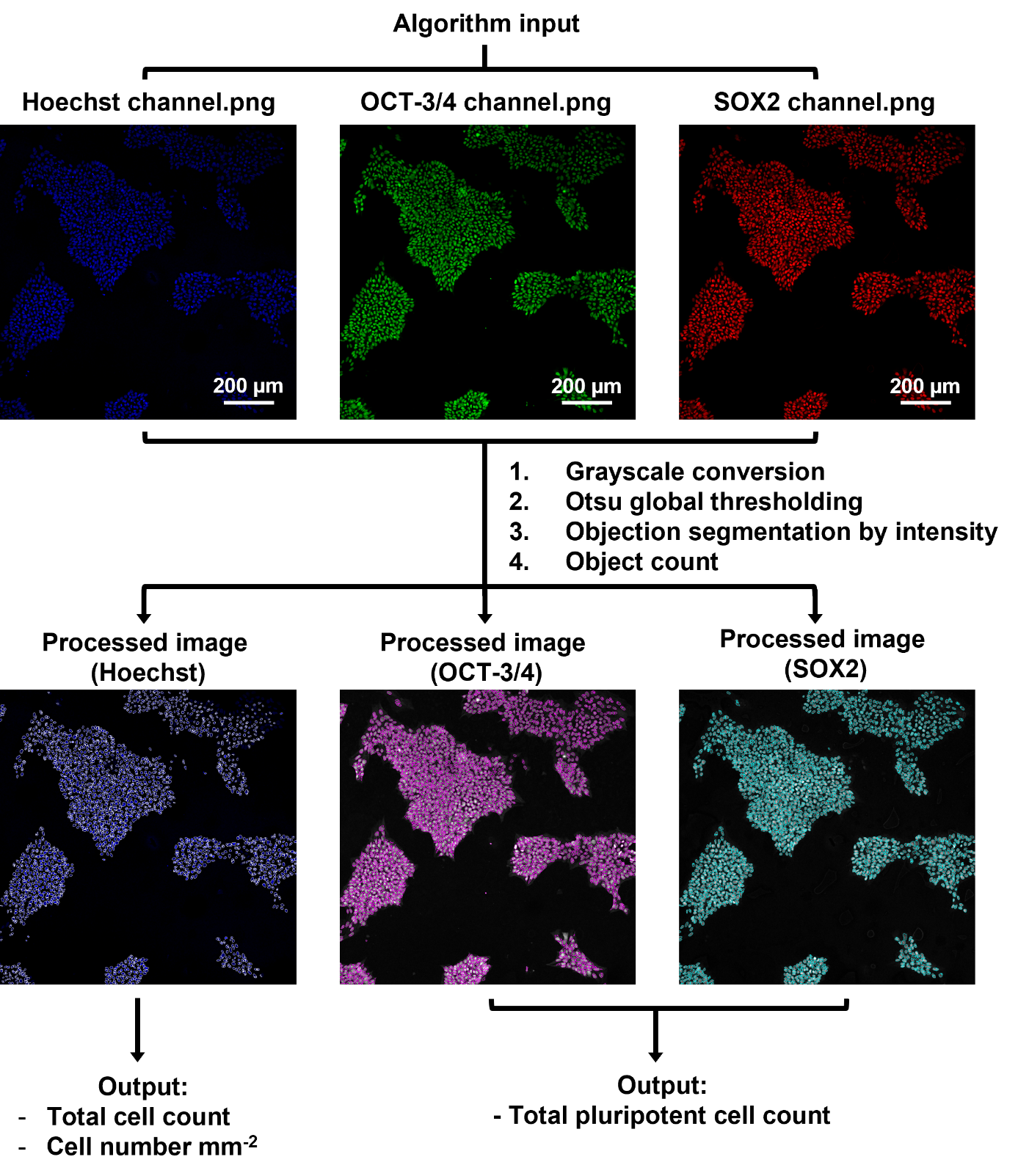


**Figure S2. Cell Profiler pipeline workflow used to analyze the output from the fractional factorial screening.** Immunofluorescent microscopy images of hiPSCs on screening materials were used as input parameter for the Cell Profiler pipeline. Images were processed to extract the total cell count per image and the related percentage of cells that remained pluripotent.

**Table S1. Composition of the 32 conditions used in the fractional factorial screening of the BU-additive library.**


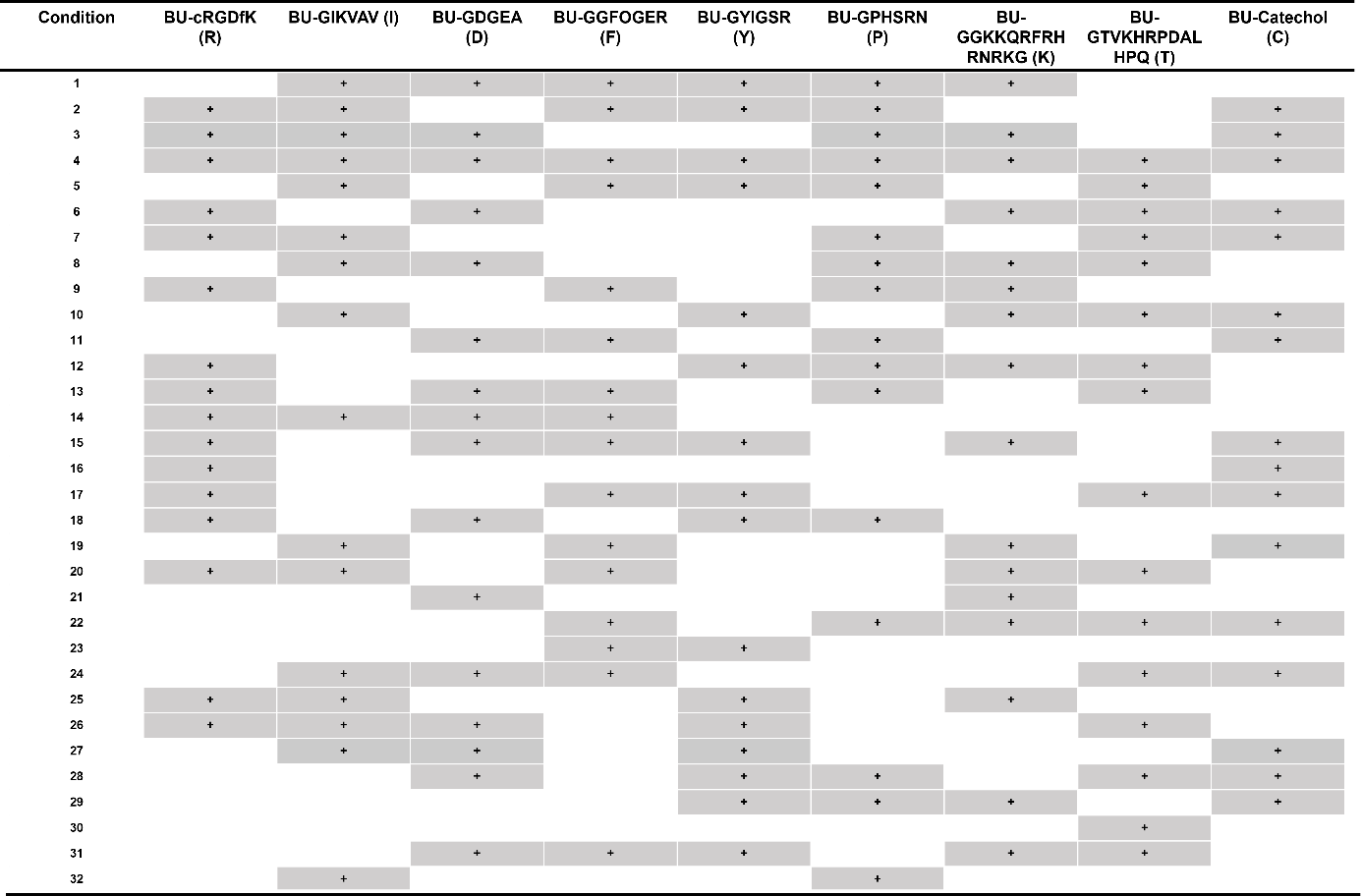


**Table S2. Overview of antibodies used for histochemical techniques.**


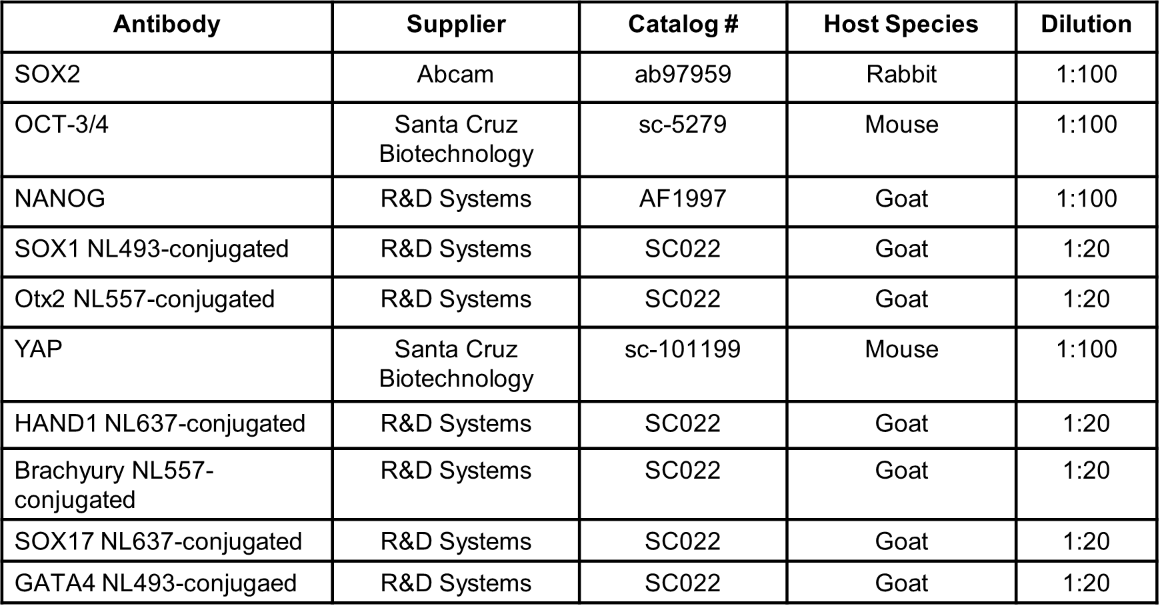


**Synthesis of bisurea-peptide additives**

*Synthesis of BU-GKKQRFRHRNRKG and BU-GTVKHRPDALHPQ*

All chemicals were purchased from Sigma-Aldrich unless otherwise stated. Peptides were synthesized using standard Fmoc solid-phase peptide synthesis on a rink amide resin at 0.1 mmol scale. The Fmoc-group was removed from Fmoc-Gly-Gly-Lys(Boc)-Lys(Boc)-Gln(Trt)-Arg(Pbf)-Phe-Arg(Pbf)-His(Trt)-Arg(Pbf)-Asn(Trt)-Arg(Pbf)-Lys(Boc)-Gly (178 mg, 0.05 mmol), and Fmoc-Gly-Thr(tBu)-Val-Lys(Boc)-His(Trt)-Arg(Pbf)-Pro-Asp(OtBu)-Ala-Leu-His(Trt)-Pro-Gln(Trt) using 20 v/v% piperidine in DMF mixture for 2x15 min, while still on the rink amide resin. A BU-synthon (108 mg; 0.1 mmol) (SyMO-Chem) was dissolved in 4 mL DMF. DIPEA (44 μL, 0.25 mmol) and HATU (38 mg, 0.1 mmol) were dissolved in 2 mL DMF, respectively, added to the BU-synthon solution, and stirred for 30 min. After pre-activation, the reaction mixture was added to peptides on resin and reacted overnight under shaking conditions at room temperature. The BU-peptides were cleaved from the resin in a 95:2.5:2.5 v/v% TFA:TIS:H_2_O mixture for 2 hours. The cleavage mixture was precipitated 3x in ice-cold Et_2_O. The precipitate was collected, dissolved in a mixture of water/acetonitrile, and freeze-dried. The resulting crude compounds were purified using preparative reverse phase LC-MS on a C_18_ column using gradients of acetonitrile in H_2_O, containing 0.1% TFA. Final yields after purification: 35 mg BU-GGKKQRFRHRNRKG (24%), and 25 mg BU-GTVKHRPDALHPQ (28%) as fluffy white solids after freeze-drying. All BU-peptide additives had a purity of >99%. BU-GGKKQRFRHRNRKG (C_124_H_227_N_39_O_33_): calcd. MW = 2792.42 g mol^-1^, LC-MS(ESI) t_r_ = 6.84 min, m/z found: 699.3 [M+4H]^4+^, 931.3 [M+3H]^3+^, 946.3 [M+H+2Na]^3+^, 1397.0 [M+2H]^2+^. BU-GTVKHRPDALHPQ (C_115_H_204_N_28_O_34_): calcd. MW = 2523.06 g mol^-1^, LC-MS(ESI) t_r_ = 7.1 min, m/z found: 505.4 [M+5H]^5+^, 631.9 [M+4H]^4+^, 856.4 [M+H+2Na]^3+^, 1262.3 [M+2H]^2+^, 1273.4 [M+H+Na]^2+^, 1284.8 [M+2Na]^2+^.

*Synthesis of BU-GIKVAV*

The Gly-Ile-Lys(ivDde)-Val-Ala-Val peptide was synthesized using standard Fmoc solid-phase peptide synthesis on a rink amide resin at 0.1 mmol scale. The Fmoc-group was removed from Fmoc-Gly-Ile-Lys(ivDde)-Val-Ala-Val (194 mg, 0.245 mmol) using 20 v/v% piperidine in DMF mixture for 2x15 min, while still on the rink amide resin. The peptide was cleaved from the resin in a 95:2.5:2.5 v/v% TFA:TIS:H_2_O mixture for 2 hours. The cleavage mixture was precipitated 5x in ice-cold Et_2_O and subsequently freeze-dried to yield a white fluffy solid. The Gly-Ile-Lys(ivDde)-Val-Ala-Val -NH_2_ peptide (79 mg, 0.1 mmol) was dissolved in 2 mL DMF. A BU-synthon (54 mg; 0.05 mmol) was dissolved in 4 mL DMF. DIPEA (87 μL, 0.5 mmol) and HATU (22.8 mg, 0.06 mmol) were dissolved in 2 mL DMF, respectively, added to the BU-synthon solution, and stirred for 30 min. After pre-activation, the reaction mixture was added to the peptide solution and reacted overnight under shaking conditions at room temperature. The reaction was stopped by precipitating the mixture 3x in ice-cold Et_2_O. The resulting precipitate was collected and freeze-dried into a white fluffy solid. The BU-GIK(ivDde)VAV additive was deprotected using 6 mL of 2 v/v% hydrazine monohydrate in DMF mixture under stirring conditions at room temperature for 30 min. The mixture was precipitated 3x in ice-cold Et_2_O and afterwards freeze-dried into a white fluffy solid yielding 74 mg crude BU-GIKVAV (89%). The resulting crude compound was purified using preparative reverse phase LC-MS on a C_18_ column using a gradient of 45-50% of acetonitrile in H2O, containing 0.1% TFA, resulting in a purity >99%. The final yield after purification was 34 mg BU-GIKVAV (36%). BU-GIKVAV (C_79_H_153_N_13_O_23_): calcd. MW = 1652.12 g mol^-1^, LC-MS(ESI) t_r_ = 4.19 min, m/z found: 551.83 [M+3H]^3+^, 827.08 [M+2H]^2+^, 1674.92 [M+Na]^+^.


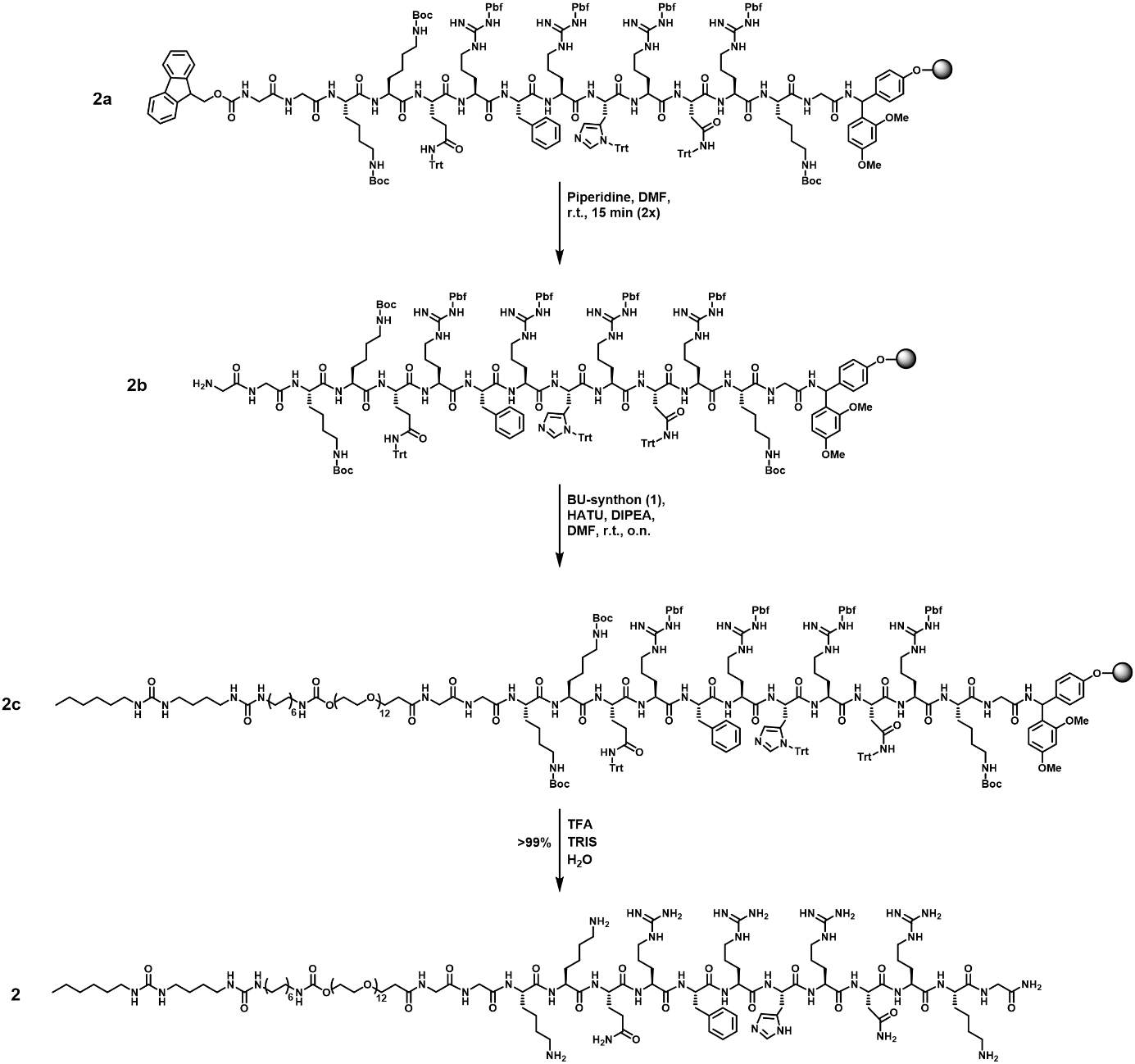


**Scheme S1.** Synthesis route for BU-GGKKQRFRHRNRKG. Reaction conditions are depicted in the scheme.


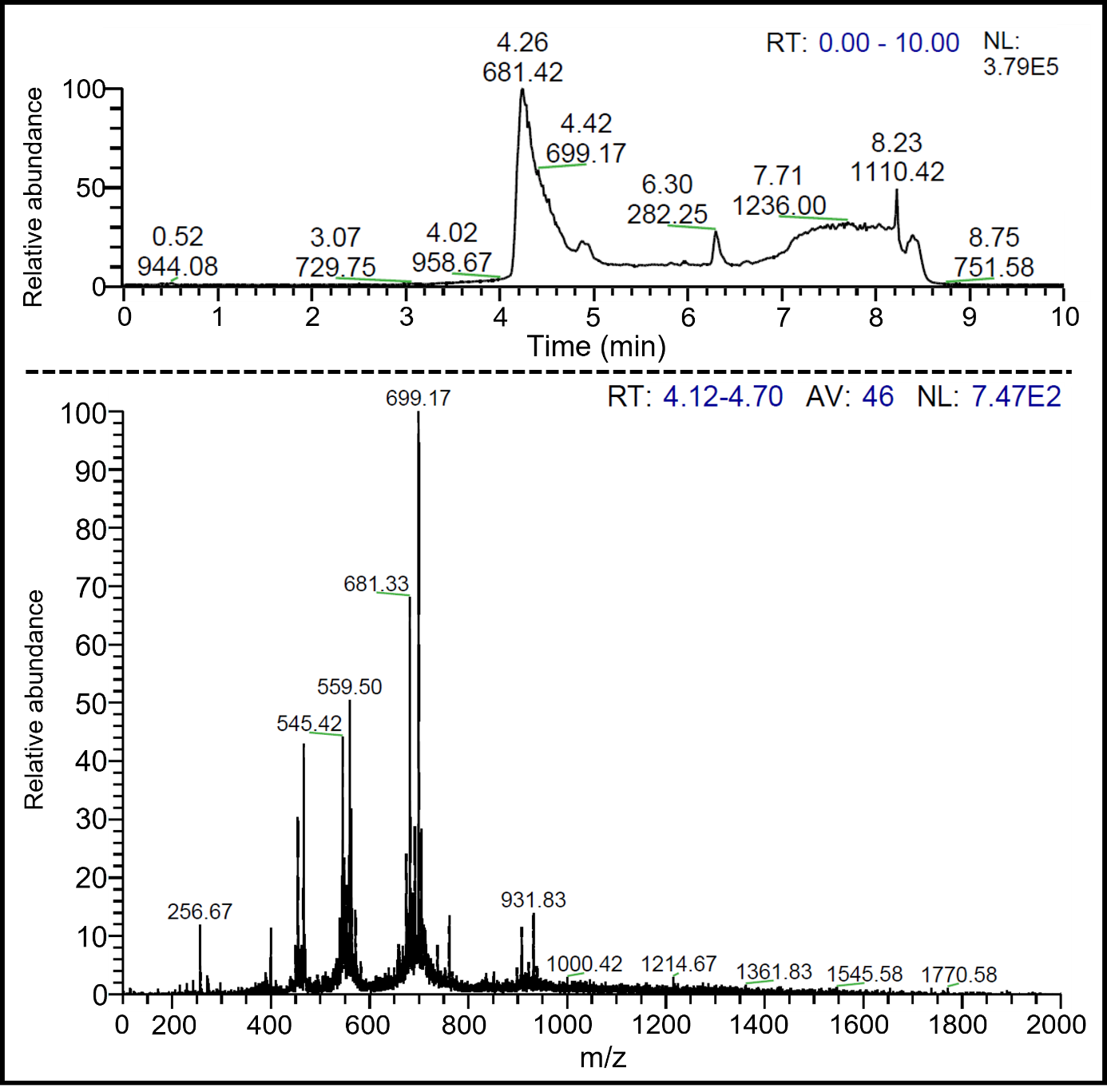


**Figure S4.** LC-MS (ESI) spectrum of BU-GGKKQRFRHRNRKG.


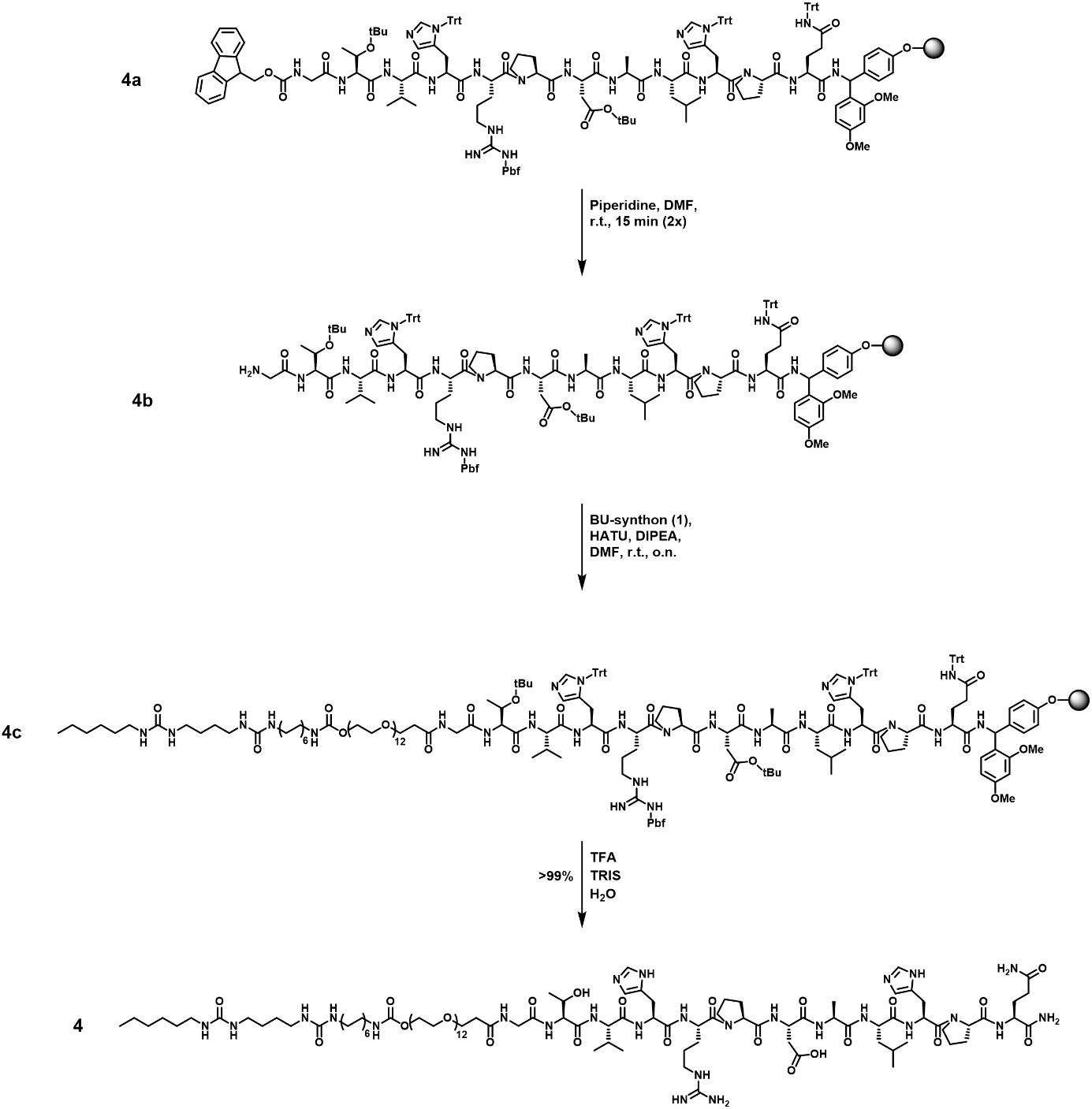


**Scheme S2.** Synthesis route for BU-GTVKHRPDALHPQ. Reaction conditions are depicted in the scheme.


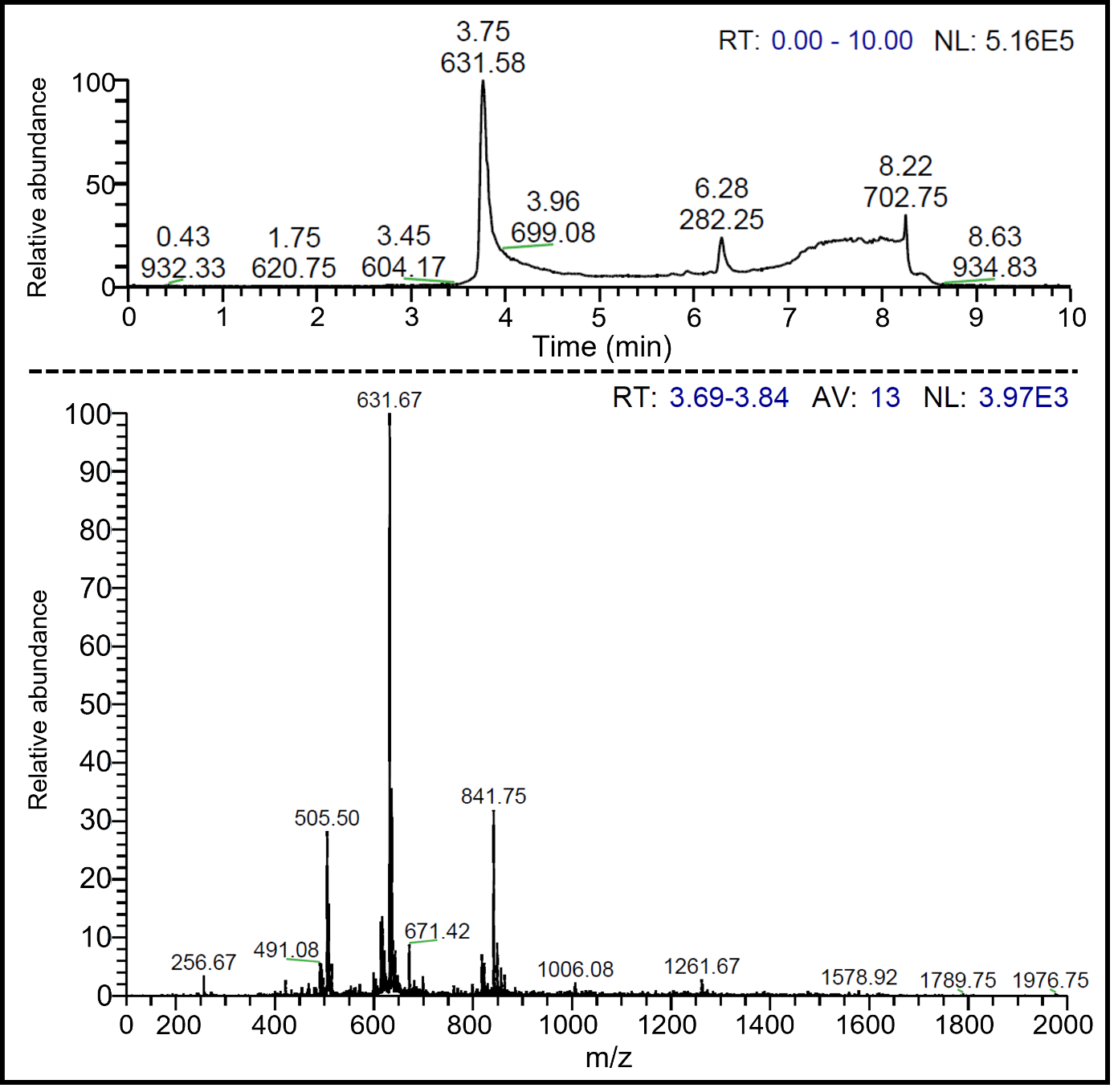


**Figure S5.** LC-MS (ESI) spectrum of BU-GTVKHRPDAHLPQ.


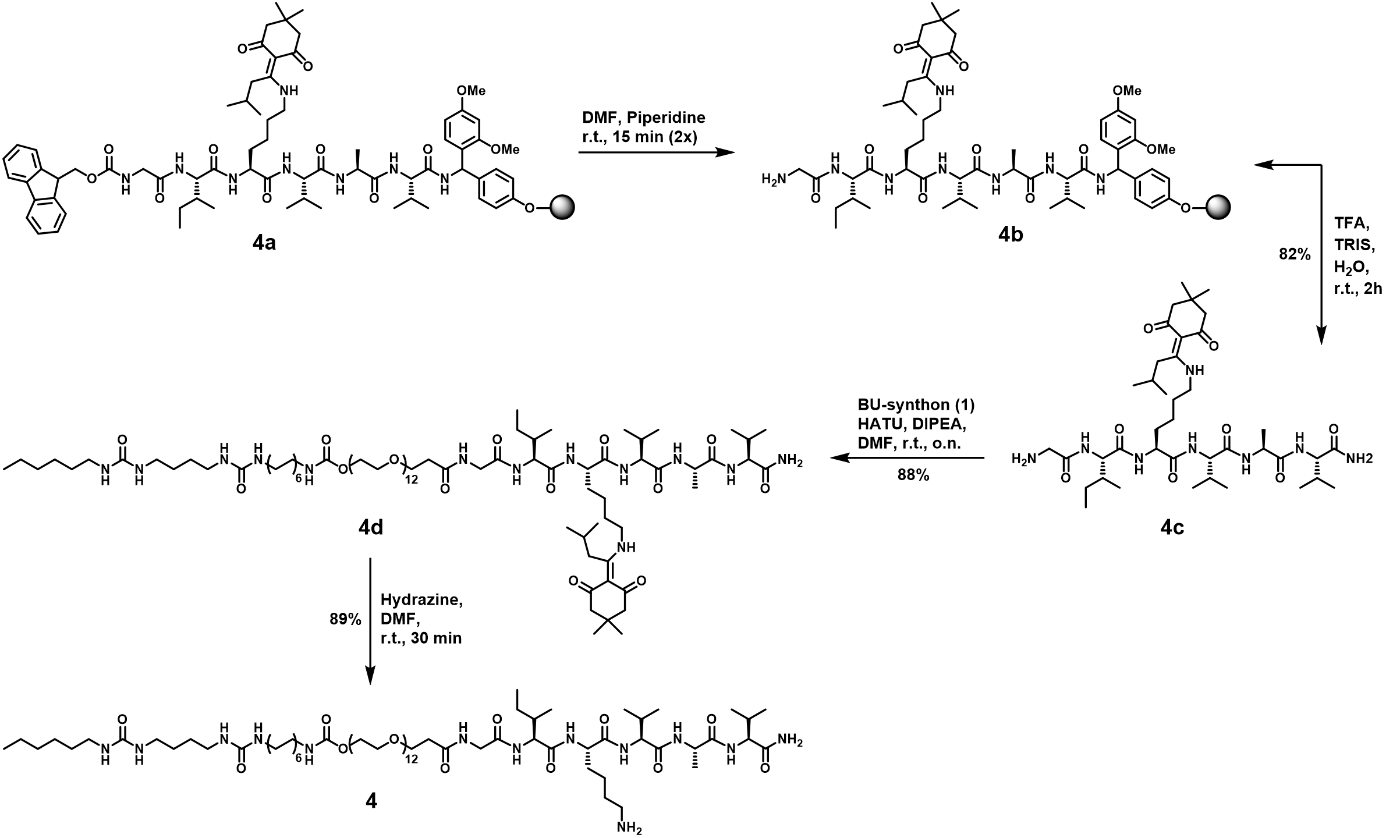


**Scheme S3.** Synthesis route for BU-GIKVAV. Reaction conditions are depicted in the scheme.


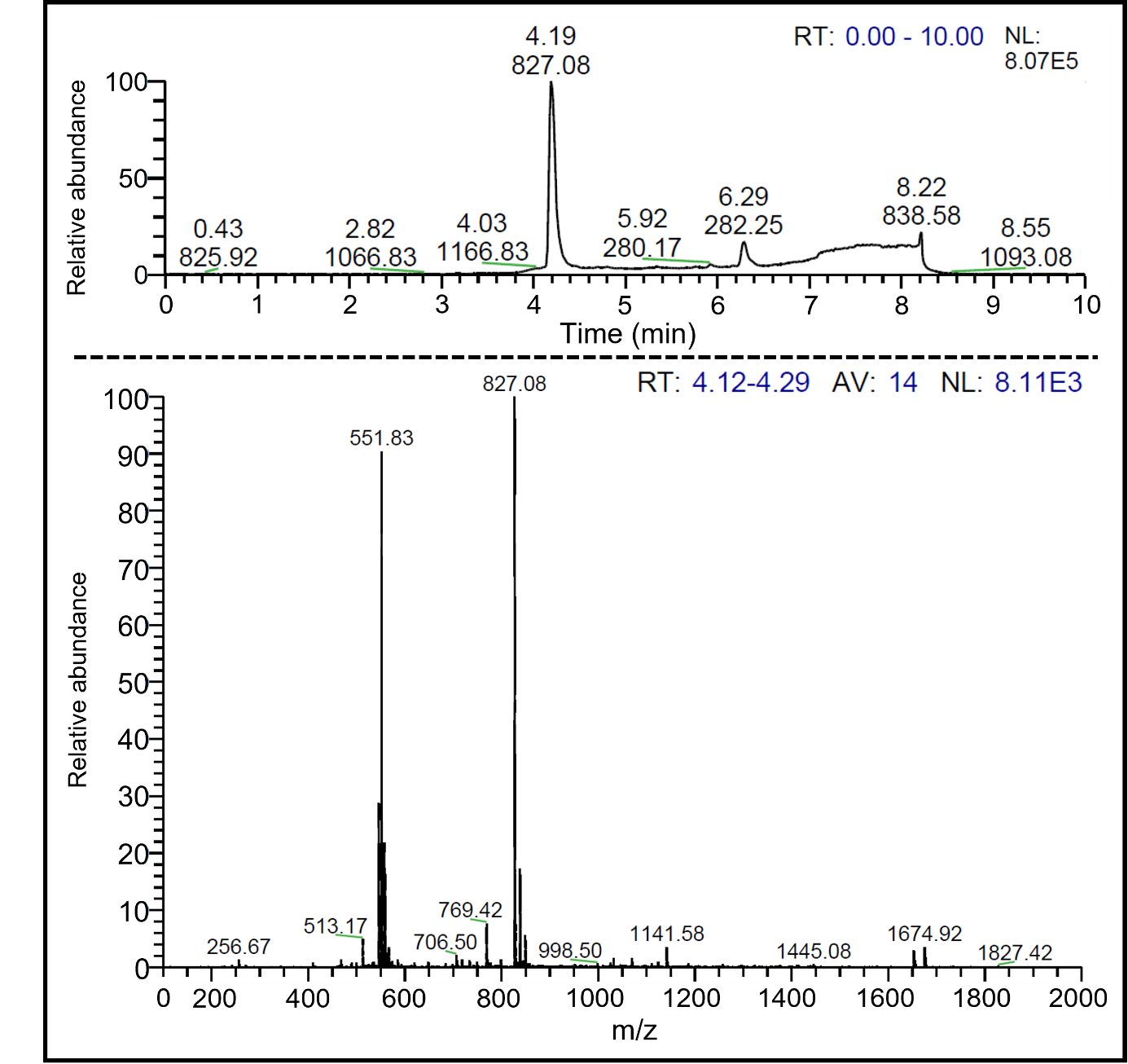


**Figure S6.** LC-MS (ESI) spectrum of BU-GIKVAV.
